# Balancing the rota: registered nurses’ preferences for shift patterns – a discrete choice experiment

**DOI:** 10.1136/bmjopen-2026-116968

**Published:** 2026-08-18

**Authors:** Chiara Dall’Ora, Hannah Ruth Barker, Talia Emmanuel, Ourega-Zoé Ejebu, M Jamal Hossain

**Affiliations:** 1 School of Health Sciences, University of Southampton, Southampton, UK; 2 NIHR Applied Research Collaboration Wessex, Southampton, UK

**Keywords:** Organisation of health services, HEALTH ECONOMICS, Nurses

## Abstract

**Abstract:**

**Objective:**

To elicit and quantify registered nurses’ (RNs’) preferences for key shift attributes and to estimate the trade-offs between shift pattern characteristics and pay.

**Design:**

Cross-sectional online discrete choice experiment with d-efficient design; 12 choice tasks per respondent. Preferences were estimated using multinomial logit models with clustered standard errors; willingness to accept (WTA) values were derived as ratios of attribute coefficients to the pay coefficient.

**Setting:**

United Kingdom.

**Participants:**

1449 registered nurses (RNs) recruited via the Nursing and Midwifery Council distribution list.

**Primary and secondary outcome measures:**

Utility coefficients (β) for shift length, schedule predictability, shift request flexibility, weekend working frequency, pay level and night-shift frequency; WTA derived from the pay attribute; subgroup and interaction analyses by age, pay band, current shift status, caring responsibilities, sector and satisfaction.

**Results:**

All six attributes were statistically significant predictors of shift pattern choices. The strongest aversions were to night-only working (β=−1.113) and having no whole weekends off (β=−0.790), which required an estimated 29% additional compensation in the primary model and 21% additional compensation, respectively. Unpredictable rotas (β=−0.220), restricted shift-choice flexibility (β=−0.189 for only swaps) and absence of pay increase (β=−0.525) also reduced utility. Nine-to-ten-hour shifts were preferred over 8-hour shifts (β=0.144), whereas 12.5-hour shifts were disliked (β=−0.179). Subgroup analyses showed significant heterogeneity by age and current shift status, with more limited evidence of pay-band differences. Interaction analyses suggested broadly consistent aversion to night work and weekend loss across the nursing workforce.

**Conclusions:**

Nurses strongly prefer rota designs that avoid exclusive night work and protect whole weekends off. Predictability and meaningful scope to request shifts also matter. These preference estimates provide policy-relevant benchmarks for rota design and unsocial-hours compensation.

**STRENGTHS AND LIMITATIONS OF THIS STUDY**

- Large national discrete choice experiment (n=1449) translating preferences into policy-ready willingness-to-accept benchmarks for unsocial hours.
- Comprehensive attribute set (nights, weekends, shift predictability, shift length, shift flexibility, pay) selected from literature and stakeholder interviews; robust clustered multinomial logit estimation.
- Subgroup and interaction analyses probe heterogeneity (age, seniority, current shift status), improving applicability of findings.
- Stated preferences may not perfectly predict behaviour; sample skewed towards National Health Service and older registered nurses; attributes specified categorically (dose–response not modelled).

## Introduction

 Health systems internationally and in the UK face persistent nursing workforce challenges.^1–3^ Registered nurse (RN) shortages and turnover reduce continuity of care, increase costs associated with temporary staffing, and place sustained pressure on remaining staff.^4–7^ While the determinants of recruitment and retention are multi-faceted,^8^ shift patterns are both relevant to nurses and amenable to organisational change. Shift work is structurally required to deliver 24-hour care, yet shift work characteristics such as extended shift length (12 hours or longer), frequent nights and rotating patterns carry substantial personal and professional costs.^9–11^ Night work disrupts circadian rhythms and sleep architecture,^12^ and weekend duties constrain family and social time. Rotas that are difficult to predict create planning burdens for childcare or any caring commitments, education and community participation.^13^ Conversely, predictable, equitable and participatory scheduling systems can improve the experience of work and support retention.^14–16^ The policy environment for nursing work in the UK is shaped by Agenda for Change terms and conditions, including unsocial hours enhancements for nights, weekends, bank holidays, as well as by local policies governing schedule construction, predictability and opportunities for staff input.^17^ Although recent National Health Service (NHS) workforce policies have increasingly recognised the importance of flexible working and staff experience,^18 19^ relatively little quantitative evidence exists on how nurses value specific rota characteristics, such as predictability, flexibility and weekend working, or the trade-offs they are willing to make between these factors and pay. Evidence to support the enhancement and design of scheduling systems remains uneven, with many decisions made pragmatically to meet service targets.^15^ Evidence to inform systematic decisions that align organisational needs with what nurses value most is needed.

Qualitative studies have described nurses’ lived experience of shift patterns, highlighting the importance of predictability, blocks of whole-weekend time off and meaningful input into scheduling,^13 20^ but they offer limited insight on how much each shift characteristic matters relative to others. In particular, the literature does not quantify the trade-offs nurses would accept between, for example, night frequency, weekend working, schedule predictability, shift length and pay. Understanding these trade-offs is important because current evidence suggests that pay dissatisfaction is associated with intentions to leave the NHS,^21^ and that younger nurses in particular report lower pay satisfaction than their more senior colleagues.^22^ Yet it remains unclear whether financial incentives are sufficient to compensate for undesirable rota characteristics, or which aspects of shift work nurses value most highly relative to pay. Discrete choice experiments (DCEs) are well suited to quantifying these monetary and non-monetary trade-offs simultaneously, because they allow estimation of the relative importance of multiple attributes and derivation of marginal rates of substitution including willingness to accept (WTA) monetary compensation for non-monetary working conditions.^23^

The aim of this study is therefore to elicit and quantify RNs’ stated preferences for key shift pattern attributes and their associated trade-offs, and to derive WTA estimates that can inform rota design and enhancement policies. A secondary aim was to examine whether preferences vary across key workforce subgroups including age, seniority and current shift status, on the basis of the a priori hypothesis that these characteristics are likely to shape the disutility of unsocial working conditions.

## Methods

### Study design and attribute development

We designed a DCE in which respondents chose between pairs of hypothetical shift patterns. DCEs are rooted in the random utility theory and assume that each option has an overall ‘value’ (utility) to a person made up of the attributes that are presented to them plus unobserved influences; people tend to choose the option with the highest overall value, so choices are predicted as probabilities rather than certainties. This framework allows DCEs to estimate how strongly each attribute drives choice and quantify trade-offs.^23 24^ This approach has been increasingly applied in health economics to understand patient preferences,^25^ but fewer studies have examined healthcare worker preferences for employment conditions,^26–28^ and none on shift patterns that we are aware of. We referred to Li and colleagues’ methodological review of DCEs in nursing workforce research to ensure reporting rigour in line with their recommendations,^29^ and report our findings according to the DIRECT checklist.^30^

Each shift pattern was defined by six attributes that are commonly varied within nursing rotas and directly experienced by staff. Attributes were selected through a scoping of literature,^13 20^ and a qualitative interview study of 28 RNs, ward managers and service directors in five NHS Trusts across England.^15^ Data saturation was assessed iteratively throughout data collection and analysis. No new shift-pattern attributes or concepts relevant to rota preferences were identified during the final interviews. This approach is consistent with recommendations for qualitative attribute development in DCEs.^31^ Our aim was to include attributes that managers can feasibly adjust, that are relevant to nurses, that capture the main trade-offs inherent in shift work and that have the capacity to be traded. During attribute development, we considered whether candidate attributes were salient to participants, amenable to organisational change and capable of being traded against alternative rota characteristics within realistic work scenarios. Tradeability was assessed during qualitative interviews and pilot testing by exploring participants’ interpretations of the attributes and whether they considered them when making choices between hypothetical shift patterns. No substantial concerns regarding attribute relevance, overlap or inability to make trade-offs were identified. The potential for conceptual overlap between shift predictability and shift requests was carefully considered. Shift predictability refers to how far in advance shift schedules are communicated, while shift requests refer to the capacity to influence which shifts are assigned. In the qualitative phase, participants consistently distinguished between these two dimensions when discussing their working conditions: nurses described predictability as knowing what was coming, and requesting as having a say in shaping it. Both were retained as conceptually and practically distinct attributes.^15^

Table 1 reports the list of attributes and levels we designed. Salary levels are expressed relative to the respondent’s current/main job pay in 10% increments (0%, +10%, +20%).

**Table 1:** Attributes and levels used in the discrete choice experiment

Attribute	Level 1	Level 2	Level 3
Shift length	8 hours or less	Between 9 and 10 hours	12.5 hours or longer
Shift predictability	Predictable shift pattern	Sometimes predictable shift pattern	Unpredictable shift pattern
Shift requests	Only swaps with colleagues	Two choices a month	Between 3 and 4 choices a month
Weekends off	No whole weekends off	One whole weekend off per month	At least two whole weekends off per month
Salary	No change in pay from main job	+10% in pay from main job	+20% in pay from main job
Night work	No night shifts	A mix of day and night shifts	Night shifts only

Night shifts are defined as any shift that includes at least 3 hours of work between 23:00 and 06:00, consistent with standard National Health Service Agenda for Change terms.

We included shift length with three levels to evaluate trade-offs between short, mid-length and very long duties. The evidence questions the value propositions of ≥12-hour shifts (fatigue, productivity/safety concerns),^9^ so estimating preferences for alternatives is policy-relevant. A 9–10 hours option was added because DCEs are designed to test plausible alternatives that may not yet be widespread, and recent research found that, while seldom worked, 9–10 hours was the ideal shift length for 45% of the 832 RNs in the sample.^13^ Shift predictability was selected because our interviews uncovered that predictable scheduling enables planning for rest and caring responsibilities and is viewed by staff and managers as pivotal to well-being and retention.^15^ In terms of shift requests, graduated levels reflect feasible increases in scheduling autonomy, which the evidence links to better adaptation to shift work and higher satisfaction.^20^ Regular, guaranteed weekends off are consistently valued and help recovery and family life^15 20^; the levels capture meaningful steps organisations can implement. Night exposure is a core determinant of fatigue and satisfaction^13 15 20^; comparing none, mixed and nights-only patterns quantifies aversion to nights. Pay provides a monetary anchor to estimate WTA compensation for less desirable rota features, following established DCE practice in health-workforce research.^27^ Pay band refers to NHS Agenda for Change salary band.

Overall, the specification translates prominent scheduling issues into policy-testable levers (predictability, autonomy, recovery time, night exposure) and monetises the trade-offs nurses are willing to make among them.

**Figure 1:**
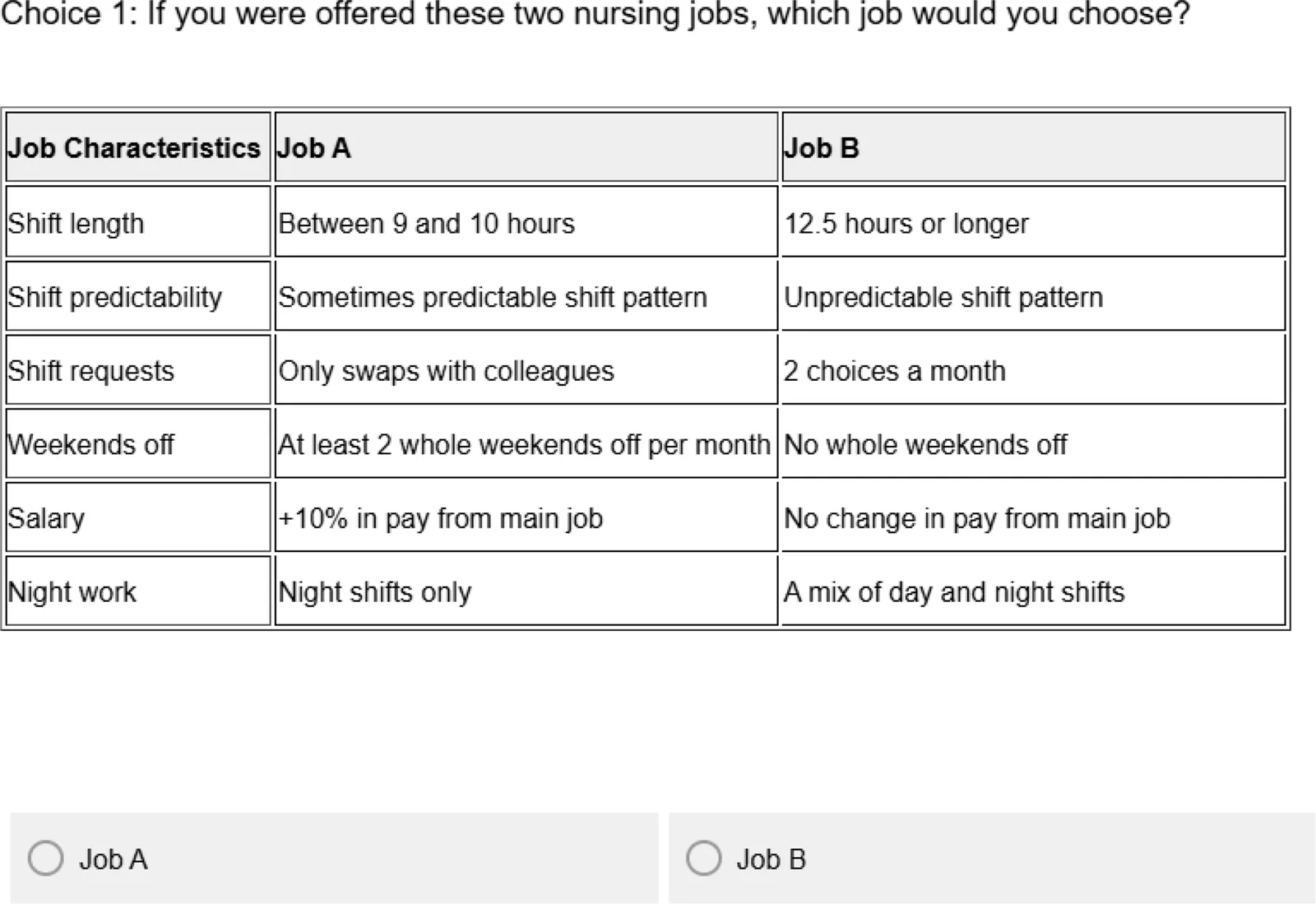
Example choice task.

We used Ngene experimental software to generate the design.^32^ We used a fractional factorial D-efficient design with six attributes, each with three levels (3^6^) to generate 24 shift pattern choices. We did not specify any priors. Choice tasks used a forced-choice format between two unlabelled alternatives, with no opt-out or status quo option. To reduce cognitive burden among respondents, they were blocked into two versions. Specifically, each participant completed 12 choice tasks, each presenting two jobs with shift pattern alternatives with different combinations of attributes and levels. We checked the designs to ensure they met the required criteria, including orthogonality, level balance with minimum overlap and utility balance.^33 34^ The design estimated main effects. The DCE was integrated into a more comprehensive survey that included brief instructions, practice items and demographic questions to characterise respondents’ employment context, including nation, sector, pay band, age, caring responsibilities, current shift work status and commute time. For an example choice task, see figure 1.

### Participant and settings

Eligibility required current employment as an RN in any UK setting. Although qualitative work to identify attributes was undertaken with nurses working in England, the final attributes (shift length, predictability, ability to request shifts, weekends off, night work and pay expressed as a percentage change) reflect core features of rota design that are common to NHS employers.

### Data collection

We created an online version of the survey on Qualtrics. Participants provided informed consent by completing an online checkbox at the end of the Participant Information Sheet prior to commencing the survey. The Nursing and Midwifery Council (the nursing and midwifery regulator in the UK) shared the survey to approximately 50 000 registrants, aiming to achieve at least a 1.5% response rate. The survey was open between July and August 2025. There were no financial incentives for participating in the survey. We used Louviere’s formula accounting for the number of DCE choice tasks, alternatives and levels to estimate the minimum sample size of 63.^35^ After 50 RNs completed a pilot survey, no substantial issues were identified regarding task comprehension, wording, attribute interpretation, survey timing or respondent burden. No substantive changes were made following piloting. A total of 1449 RNs completed the survey. The median response time was 7 min and 12 s.^36^

### Statistical analysis

The random utility maximisation framework^37^ was used to explore the data, as it is assumed that in each shift pattern task (t=1,…,T), nurses (n=1,…,N) derive utility (satisfaction) U_njt for a job (j=1,…,J). Then, they choose to accept the shift pattern alternative if the associated utility exceeds the utility of the other shift pattern alternative on offer. The utility of the shift pattern alternative (ie, dependent variable) is a combination of the deterministic (ie, observables) and random (unobservable) components.

Choice data were analysed using multinomial logit (MNL) models. MNL was selected as the primary model because the objective was to estimate transparent, policy-interpretable average preferences and WTA benchmarks. Observed heterogeneity was explored through prespecified subgroup and interaction analyses. We acknowledge that MNL assumes independence of irrelevant alternatives and homogeneous unobserved preferences; mixed logit and latent class models would relax these assumptions and represent a valuable extension, but were beyond the policy-focused scope of the present analysis.

The choice alternatives were unlabelled generic shift patterns, presented as job A and job B. An alternative-specific constant (ASC) for job A was included as a position constant to assess any systematic preference for one presented alternative over the other that was not explained by the observed attributes. The models estimated coefficients (β) for each non-reference attribute level, representing the utility associated with that level relative to the reference category. For each attribute, the highest-utility level served as the reference category (coded as 0), allowing other levels to be interpreted as utility decrements relative to the most preferred option.

The MNL model assumes independence of irrelevant alternatives and homogeneous preferences across respondents. Coefficients represent the marginal utility of each attribute level relative to the reference, allowing comparison of relative importance across attributes. SEs were estimated using robust variance-covariance matrices to account for panel structure (multiple choice observations per respondent). We derived WTA compensation measures using the pay attribute as the monetary numeraire. WTA for a given attribute level was computed as the ratio of the attribute coefficient to the average pay coefficient (across ‘no change’ and ‘+10%’), yielding values in units of pay steps of approximately 10 percentage points. Averaging across pay coefficients provided a stable monetary numeraire given the categorical nature of the pay attribute. Positive WTA values indicate additional compensation required to accept the non-reference level; negative WTA values indicate a preference for the non-reference level relative to the reference. CIs for WTA estimates were derived using the delta method. Subgroup analyses explored heterogeneity by age groups, pay bands, current shift status, satisfaction levels, caring responsibilities and sector. Subgroup and interaction analyses were exploratory and were not adjusted for multiple comparisons; therefore, marginal findings, particularly those close to p=0.05, should be interpreted cautiously. We also ran simple task-position checks to identify potential learning or fatigue effects, including a linear trend term and attribute-by-task interactions for the most influential attributes. Model diagnostics included examination of coefficient signs and magnitudes, CI widths and information criteria (Bayesian Information Criterion (BIC), Akaike Information Criterion (AIC)). All analyses were conducted in R, V.4.5.0^38^ and the apollo package.^39^

### Patient and public involvement

Patients or the public were not involved in the design, conduct, reporting or dissemination plans of this research. The study surveyed RNs.

## Results

The final sample comprised 1449 RNs, yielding a response rate of 2.9%. No respondents were excluded after completing the DCE tasks. Geographic distribution was 77.4% England, 13.5% Scotland, 5.9% Wales and 2.9% Northern Ireland. Most respondents worked in the NHS (84.5%). Pay bands were concentrated in bands 5–7 (40.2%, 30.0% and 19.9%). The mean age was 49.8 years (SD 12.4). At the time of survey, 72.4% were working shifts. Among shift workers, weekend duty frequency was typically one or two weekends per month and the mean number of night shifts per month was 2.6 (SD 4.0).

Table 2 presents comprehensive descriptive statistics for the sample, organised by demographic characteristics, employment characteristics and current working patterns.

**Table 2:** Descriptive statistics

Characteristic	Category/measure	N	%	Mean (SD)
Geographic distribution				
	England	1122	77.4	
	Scotland	195	13.5	
	Wales	85	5.9	
	Northern Ireland	42	2.9	
Sector				
	NHS	1215	84.5	
	Other healthcare provider	174	12.0	
	Social care	49	3.4	
Pay band				
	Band 5	582	40.2	
	Band 6	435	30.0	
	Band 7	288	19.9	
	Band 8a–8d	133	9.2	
	Band 9	4	0.3	
	Other	7	0.5	
Age				49.8 (12.4)
	21–30 years	122	8.5	
	31–40 years	250	17.5	
	41–50 years	281	19.7	
	51–60 years	444	31.1	
	61+ years	330	23.1	
Caring responsibilities				
	Has childcare responsibilities	495	34.2	
	Number of children (if any)			1.8 (0.9)
	Has adult caring responsibilities	409	28.3	
	Any caring responsibilities	891	61.5	
Current shift work				
	Currently working shifts	1049	72.4	
	Considering shift work (non-shift workers)	162	42.0	
	Shift length (hours)			10.9 (4.6)
	Weekly contracted hours			30.7 (16.3)
	Bank hours per month			5.5 (14.3)
Weekend working (current shift workers)				
	One weekend per month	382	31.3	
	Two weekends per month	469	38.4	
	Three weekends per month	284	23.2	
	Four weekends per month	87	7.1	
Night working (current shift workers)				
	Number of night shifts per month			2.6 (4.0)
Schedule predictability				
	Unpredictable	570	39.5	
	Sometimes predictable	304	21.0	
	Predictable	571	39.5	
Shift choices/requests				
	Only swaps with colleagues	283	19.8	
	1–2 choices per month	284	19.9	
	3–4 choices per month	352	24.6	
	5+ choices per month	226	15.8	
	No shift choices	284	19.9	
Current satisfaction				
	Very dissatisfied	129	8.9	
	Moderately dissatisfied	257	17.8	
	Moderately satisfied	604	41.8	
	Very satisfied	456	31.5	
Commute time				
	0–15 min	380	26.2	
	15–30 min	579	40.0	
	30–60 min	389	26.9	
	Over 60 min	100	6.9	

Percentages are calculated on available (non-missing/based on valid responses) data, as denominators vary across characteristics. Missing data: age (n=22), childcare (n=1), adult caring (n=4), shift length (n=12), weekly hours (n=14), bank hours (n=31), night shifts (n=20), predictability (n=4), shift choices (n=20), satisfaction (n=3), commute (n=1). Pay band refers to NHS Agenda for Change salary band. Bank hours per month refers to additional paid shifts undertaken through staff bank arrangements, beyond contracted hours.

NHS, National Health Service.

### Baseline MNL estimates

Table 3 summarises the baseline MNL estimates for the main attributes and levels relative to their references. The coefficients reported are MNL utility weights; each reflects the estimated change in utility associated with a given attribute level relative to the reference category (the most preferred level for each attribute). The ASC for job A was small and not statistically significant (β=0.03, p=0.06), indicating limited evidence of systematic position bias.

**Table 3:** Multinomial logit estimates

Attribute	Level	Coefficient (β)	SE	95% CI lower	95% CI upper
Shift length	9–10 hours	0.144*	0.027	0.092	0.197
Shift length	12.5 hours	−0.179*	0.030	−0.238	−0.120
Predictability	Unpredictable	−0.220*	0.021	−0.261	−0.179
Shift requests	Two choices/month	−0.098*	0.022	−0.142	−0.054
Shift requests	Only swaps	−0.189*	0.024	−0.236	−0.143
Weekend working	One weekend off	−0.280*	0.026	−0.331	−0.229
Weekend working	No weekends off	−0.790*	0.029	−0.847	−0.733
Pay	+10%	−0.232*	0.021	−0.273	−0.192
Pay	No change	−0.525*	0.028	−0.579	−0.471
Night shifts	Mix of days and nights	−0.442*	0.030	−0.500	−0.384
Night shifts	Nights only	−1.113*	0.047	−1.206	−1.020

Reference categories are 8 hours; always predictable; three to four choices/month; ≥2 whole weekends off; +20% pay; day shifts only. Negative coefficients indicate lower utility. Model fit: Akaike Information Criterion: 20 453.48; Bayesian Information Criterion: 20 522.10.

* p<0.001.

Salary, predictable schedules, greater ability to request shifts and more weekends off all increase utility. Night-only duties and 12.5-hour shifts reduce utility; mixed day/night is negative but smaller in magnitude. The 9–10-hour level is preferred to 12.5 hours and to 8 hours.

### Willingness-to-accept (

WTA values were computed using the average pay coefficient across ‘no change’ and ‘+10%’. Table 4 reports the key WTA figures in units of approximately 10 percentage points of pay.

**Table 4:** WTA estimates

Attribute	Comparison (vs reference)	WTA (pay units)	95% CI lower	95% CI upper
Night shifts	Nights only vs days only	2.938	2.607	3.269
Night shifts	Mix vs days only	1.166	0.976	1.356
Weekend working	No weekends off vs ≥2 off	2.085	1.881	2.288
Weekend working	One weekend off vs ≥2 off	0.739	0.613	0.864
Predictability	Unpredictable vs always predictable	0.580	0.476	0.685
Shift requests	Only swaps vs 3–4 choices	0.499	0.369	0.629
Shift requests	Two choices vs 3–4 choices	0.258	0.141	0.375
Shift length	12.5 hours vs 8 hours	0.472	0.320	0.625
Shift length	9–10 hours vs 8 hours	−0.381	−0.530	−0.232

Positive WTA values indicate additional compensation required to accept the non-reference level. Negative WTA indicates preference for the non-reference level. WTA expressed in units of the pay attribute (≈10% steps).

WTA, willingness-to-accept.

We observe positive WTAs for less desirable features, most notably nights-only duties and unpredictable schedules, indicating that respondents require pay increases to accept these conditions (eg, a WTA of +2.938 implies a +29.38% pay uplift for nights-only vs the reference, ie, no nights). Reductions in weekends off similarly attract positive WTAs, with the largest compensation needed when no whole weekends off are offered. Lower flexibility (eg, only swaps with colleagues) requires compensation, whereas 2 or 3–4 shift requests per month generally have negative WTAs, showing respondents would accept lower pay for greater scheduling control. For shift length, ≥12.5 hours carries a positive WTA relative to shorter days, while 9–10 hours is close to zero (or less) versus the reference, suggesting a plausible compromise. Collectively, these WTA values quantify the pay adjustments needed to offset rota burdens and the implicit value of predictability, autonomy and recovery time. Sensitivity analyses demonstrated that the WTA findings were robust to alternative specifications of the pay attribute. Recalculating WTA values using alternative pay-level coefficients resulted in some variation in absolute compensation estimates but did not materially alter the ordering of preferences. Similarly, a model specifying pay as a continuous attribute produced near-identical model fit to the primary specification (AIC 20 453.46 vs 20 453.48; BIC 20 516.81 vs 20 522.10). Across both sensitivity analyses, nights-only working and having no whole weekends off remained the two most strongly disliked job characteristics, followed by mixed day/night working. These findings suggest that the substantive interpretation of WTA estimates is robust to alternative pay specifications. Results from an additional sensitivity analysis specifying pay as a continuous attribute are presented in online supplemental tables S1a and S1b.

### Subgroup analysis

Given the diverse characteristics of the healthcare workforce, we examined whether preferences varied across key demographic and employment subgroups deemed relevant to decision-makers. Separate MNL models were estimated for subgroups defined by age, caring responsibilities, pay band, current shift status, satisfaction levels and sector. Full estimates can be found in table 5.

**Table 5:** Subgroup analysis

Subgroup	N	No whole weekends off (β)	Night shifts only (β)	No pay increase (β)	Unpredictable (β)
Full sample	1449	−0.790***	−1.113***	−0.525***	−0.220***
Age groups					
Young (<40)	372	−1.016***	−1.133***	−0.776***	−0.284***
Mid (40–54)	281	−0.827***	−1.169***	−0.550***	−0.200***
Older (55+)	757	−0.675***	−1.067***	−0.397***	−0.194***
Caring responsibilities					
Has childcare	495	−0.747***	−1.138***	−0.491***	−0.219***
No childcare	953	−0.815***	−1.103***	−0.547***	−0.222***
Any care	891	−0.793***	−1.172***	−0.484***	−0.214***
No care	554	−0.787***	−1.052***	−0.579***	−0.230***
Pay band					
Band 5 (junior)	582	−0.849***	−1.198***	−0.538***	−0.156***
Band 6–7 (mid)	723	−0.934***	−1.636***	−0.549***	−0.294***
Band 8+ (senior)	133	−1.030***	−1.971***	−0.787***	−0.312***
Current status					
Working shifts	1049	−0.725***	−0.945***	−0.531***	−0.205***
Not working shifts	384	−1.054***	−1.763***	−0.546***	−0.268***
Current satisfaction					
Currently satisfied	1060	−0.805***	−1.139***	−0.521***	−0.207***
Currently dissatisfied	386	−0.759***	−1.052***	−0.537***	−0.251***
Sector					
NHS	1215	−0.783***	−1.139***	−0.550***	−0.229***
Other sector	218	−0.826***	−0.965***	−0.399***	−0.194***

***p<0.001.

NHS, National Health Service.

Preferences vary across several workforce subgroups. While table 5 presents subgroup-specific coefficients, formal tests of coefficient equality were conducted to determine whether observed differences between groups were statistically significant (online supplemental table 2). Significant differences were observed by age and current shift status. Younger nurses were significantly more sensitive to pay reductions than older nurses (z=−5.29, p<0.001) and were more averse to having no whole weekends off (z=−4.54, p<0.001). Nurses not currently working shifts were significantly more averse than current shift workers to nights-only working (z=7.90, p<0.001) and to having no whole weekends off (z=4.25, p<0.001). Senior nurses (band 8+) were also significantly more averse to nights-only working than band 5 nurses (z=2.74, p=0.006). In contrast, although coefficients differed numerically across pay bands, formal tests did not support statistically significant differences in pay sensitivity between band 5 and band 8+ respondents (z=1.53, p=0.126). Younger workers are more pay-sensitive (nearly double the aversion to no pay rise) and place greater value on whole weekends off and predictable schedules, while aversion to night-only shifts is uniformly high across ages. Senior nurses showed significantly greater aversion to nights-only working than band 5 nurses, but differences in pay sensitivity between pay bands were not statistically significant. Non-shift workers display markedly stronger dislike of both night-only and intensive weekend work than current shift workers, consistent with selection or adaptation effects and implying that recruiting non-shift workers into shift patterns may require larger incentives. By contrast, caring responsibilities and overall job satisfaction show only modest, targeted differences (eg, slightly greater dislike of unpredictable schedules among dissatisfied staff) and sector differences indicate somewhat lower night-shift aversion and lower pay sensitivity outside the NHS.

### Interaction effects

We estimated six interaction models to explore preference moderation. Table 6 reports the interaction effects results.

**Table 6:** Interaction effects results

Interaction	Parameter	β	SE	95% CI	P value
Caring×weekend working					
	No weekends off×any care	0.011	0.054	−0.095 to 0.116	0.843
	One weekend off×any care	0.002	0.048	−0.092 to 0.096	0.969
Age×night shifts					
	**Mix days/nights×age**	**−0.009**	**0.002**	**−0.013 to −0.004**	**<0.001**
	Nights only×age	0.001	0.004	−0.006 to 0.008	0.717
Satisfaction×predictability					
	Unpredictable×satisfied	0.047	0.046	−0.043 to 0.137	0.303
	Sometimes predictable×satisfied	0.015	0.046	−0.076 to 0.106	0.751
Childcare×predictability					
	Unpredictable×childcare	0.002	0.042	−0.080 to 0.085	0.958
	Sometimes predictable×childcare	−0.060	0.042	−0.143 to 0.022	0.151
Commute×shift length					
	12.5 hour shifts×long commute	−0.126	0.064	−0.252 to −0.001	0.049
	9-10-hour shifts×long commute	0.023	0.056	−0.087 to 0.132	0.687
Pay band×pay changes					
	No pay change×band 5	−0.003	0.061	−0.124 to 0.117	0.956
	+10% increase×band 5	−0.061	0.043	−0.146 to 0.025	0.163

Statistically significant at p < 0.05.

Only two of 12 tested interactions were statistically significant; all others showed no evidence of moderation. Age×mixed day/night shifts: older workers are more averse to mixed patterns than younger workers (β=−0.009 per year, p<0.001). This implies a meaningful gradient across ages: approximately −0.26 disutility at age 30 versus −0.62 at age 70, a ~2.4-fold difference in intensity. The night-only shift interaction with age was non-significant (β=0.001, p=0.717), indicating uniformly strong aversion to exclusive night work across all ages.

Commute length×12.5 hour shifts: workers with long commutes (≥30 min) are more averse to 12.5-hour shifts than those with shorter commutes (β=−0.126, p=0.049), increasing disutility from −0.179 to −0.305. The interaction for 9–10-hour shifts was non-significant (β=0.023, p=0.687), suggesting this shift length is acceptable regardless of commute distance.

### Choice-task analysis

To assess data quality, preference stability and fatigue effects across the 12 tasks, we added task-position terms to the choice model. Nested task-position models were compared using likelihood-ratio tests and information criteria. Adding a linear task-position term improved model fit compared with the baseline model (likelihood ratio=23.67, df=1, p<0.001; AIC 20 431.81 vs 20 453.48; BIC 20 505.71 vs 20 522.10). Adding attribute-by-task interactions also improved fit relative to baseline (likelihood ratio=24.38, df=3, p<0.001), although the improvement was modest once additional parameters were penalised (AIC 20435.10; BIC 20519.55). These findings suggest a small task-position effect, but do not indicate substantial instability in preferences across the DCE. Aversion to losing whole weekends off strengthened across tasks, whereas preferences for night work and pay remained comparatively stable. This may reflect preference sharpening, fatigue or strategic updating; these mechanisms cannot be definitively disentangled with the current design.

## Discussion

This study is, to our knowledge, the first large DCE to quantify RNs’ preferences for a comprehensive set of shift pattern attributes—night work intensity, weekend work, shift length, schedule predictability, flexibility to request shifts and pay.

Our study revealed three key findings. First, nurses place greatest value on avoiding exclusive night work and protecting whole weekends off. These were substantially more important than other rota features, although predictable schedules, meaningful scope to request shifts and avoiding very long shifts also contributed to preferences. Second, subgroup analysis revealed the strongest evidence of heterogeneity by age and current shift status, with more limited evidence of pay-band differences. Third, the dominant preference pattern was highly consistent across the nursing workforce: intensive night work and loss of whole weekends were disliked across demographic and employment groups. This suggests that these rota features are core workforce concerns rather than issues confined to specific subgroups. Interaction analyses showed limited evidence of effect modification, with only two moderating effects: a gradual age-related increase in aversion to mixed day/night rotations and greater aversion to 12.5-hour shifts among staff with longer commutes. The strong aversion to intensive night work is consistent with extensive evidence that night shifts disrupt circadian rhythms and are linked to sleep disturbance, cardiometabolic risk and other adverse outcomes, including job dissatisfaction among healthcare staff.^12 40^ It is worth noting some nurses choose to work night shift only schedules due to higher pay, ability to avoid paying for childcare during the day, enjoying a calmer pace and reduced work intensity,^20^ consistent with evidence that shift preferences are driven by out-of-work priorities and by wanting to avoid in-shift fatigue.^13^ Such preferences did not surface in our sample, suggesting that such night only schedules are only preferred by a minority of the workforce.

Our finding that weekend working is strongly disliked aligns with qualitative and review evidence that unsociable hours constrain family and social life and are associated with stress and poorer work-life balance,^41 42^ all of which plausibly contribute to the high disutility for losing whole weekends. The value placed on predictable schedules and some autonomy in rostering echoes prior work showing that flexible, participatory scheduling can improve perceived control and work-life balance,^15^ even though evaluations of self-scheduling report mixed outcomes and implementation challenges.^16 43 44^ Our results suggest predictability is widely valued across demographics, which is consistent with mixed but generally positive evidence that scheduling flexibility can support satisfaction and retention if implemented equitably. The preference peak at 9–10 hours adds nuance to debates about long shifts. While many NHS services favour 12-hour long shifts based on assumptions that such long shifts improve nurse work-life balance and continuity of care,^45^ extensive evidence indicates fatigue and recovery concerns,^9^ and our data show that reducing long shifts to between 9 and 10 hours may be more acceptable to staff without reverting to shorter shifts that increase number of days at work. Finally, our WTA benchmarks can be read alongside the Agenda for Change unsocial hours framework.^46^ For most staff (bands 4–9), nights and Saturday hours attract time plus 30%, with time plus 60% on Sundays and public holidays. The primary WTA estimate for night-only patterns was approximately 29%, but sensitivity analyses using a continuous-pay specification suggested a higher estimate. Taken together, these findings indicate that current night premia may partially offset, but not necessarily fully compensate for, the disutility of sustained night working. The ~21% WTA for losing whole weekends indicates weekend enhancements are unlikely to meet the compensation level required when rosters routinely provide few or no full weekends off. This is important for early-career nurses, who appear particularly sensitive to pay and have the longest potential tenure in the NHS if retained. Although the interaction between age and mixed day/night working was statistically significant, its magnitude was small, indicating that age-related differences in preferences emerge gradually rather than representing a large practical effect at the individual level. National evidence shows that younger NHS staff report lower job satisfaction and greater dissatisfaction with pay than older colleagues, and these gaps are linked to higher intentions to leave.^21^ Policy-wise, the percentage-pay scale of our WTA estimates supports targeted, transparent premia tied to the rota features younger nurses value or find most burdensome (eg, compensation for nights-only or highly unpredictable schedules), alongside improvements in predictability and choice.

### Strengths and limitations

A key strength is the large, diverse UK sample, spanning bands, settings and regions, which strengthens generalisability. The experimental design captured the attributes most frequently cited in nursing workforce research—unsociable hours, predictability, flexibility and pay—and the analysis translated coefficients into policy-ready WTA estimates. The study also probed heterogeneity using both subgroup models and targeted interactions, and checked for learning/fatigue across tasks, enhancing confidence in preference stability.

Limitations include the inherent reliance on stated preferences, which may not perfectly predict revealed behaviour; however, DCEs are well-validated in health and are increasingly applied to workforce questions.^29^

The sample was predominantly NHS and older on average, which may limit transferability to younger segments or non-NHS employers, although our age and sector analyses showed broadly consistent preferences. In addition, attribute levels were specified categorically (eg, night pattern categories rather than counts), so non-linearities in dose–response could not be explored further. Finally, the intensification of aversion to weekend working across tasks may reflect preference sharpening, fatigue or strategic updating, and these mechanisms cannot be definitively disentangled with the current design. A formal dominance or rationality task was not included, meaning that individual-level dominated choices or non-trading behaviour could not be directly assessed.

### Implications for policy and practice

WTA benchmarks provide practical evidence for aligning rota design and unsocial-hours compensation with what nurses value. The primary WTA estimate for night-only patterns was approximately 29%, while the continuous-pay sensitivity analysis suggested a higher estimate. This indicates that current night premia may partially offset, but may not fully compensate for, the disutility of sustained night working. For weekend working, the estimated compensation required for having no whole weekends off also indicates that pay alone is unlikely to address the burden of routinely losing protected weekend time.

For employers, the clearest implication is that rota redesign should accompany financial enhancement. Limiting exclusive night patterns, protecting whole weekends where feasible, improving predictability and ensuring fair access to shift requests are likely to deliver meaningful workforce benefits. Intermediate 9–10-hour shifts may also offer a more acceptable alternative to 12.5-hour shifts, particularly where fatigue and recovery are concerns.

Policy responses should avoid relying on the assumption that some groups of nurses are simply more willing to absorb unsociable hours. Although preferences varied by age and current shift status, aversion to intensive night work and weekend loss was widespread. Sustainable retention strategies should therefore combine fair compensation with rota designs that reduce cumulative exposure to the most disliked patterns and protect recovery time.

## Conclusion

This study quantified RNs’ preferences for key dimensions of rota design using a DCE. The strongest preferences are to avoid exclusive night working and to retain whole-weekend time off, with additional value placed on predictability and meaningful scope to request shifts. Nine-to-ten-hour shifts are preferred to both 8 and 12.5 hours. Willingness to accept measures provides practical numbers for enhancement policy, but the magnitude of disutility suggests that rota redesign may deliver larger gains than pay alone. For managers and policymakers, the implications are direct: limit exclusive night patterns, guarantee intact weekends where feasible, improve predictability and request processes, and consider intermediate shift lengths. Align enhancements with measured disutility, and recognise career-stage differences when designing rotas and incentives. These steps can help build sustainable nursing teams, improve the experience of work, and thereby support safer, higher-quality care for patients.

## Supplementary material

10.1136/bmjopen-2026-116968 online supplemental table 1

10.1136/bmjopen-2026-116968 online supplemental table 2

## Data Availability

Data are available in a public, open access repository.
