# Supplementary material for: Balancing the rota: registered nurses’ preferences for shift patterns – a discrete choice experiment
Source: BMJ Open. 2026 Aug 18;16(8):e116968. doi: 10.1136/bmjopen-2026-116968 (PMC13504907; doi:10.1136/bmjopen-2026-116968)
Supplement: Supplementary file 1 [file bmjopen-2026-116968.reviewer_comments.pdf]

PEER REVIEW HISTORY

BMJ Open publishes all reviews undertaken for accepted manuscripts. Reviewers are asked to complete a checklist review form and are provided with free text boxes to elaborate on their assessment. These free text comments are reproduced below.

ARTICLE DETAILS

Title (Provisional)

Balancing the rota - registered nurses' preferences for shift patterns: a discrete choice experiment

Authors

Dall'Ora, Chiara; Barker, Hannah Ruth; Emmanuel, Talia; Ejebu, Ourega-Zoé; Hossain, M. Jamal

---

VERSION 1 - REVIEW

**Reviewer**	**1**
**Name**	**Lin, Chia-Hui**
**Affiliation**	**National Defense Medical Center School of Nursing**
**Date**	**15-Mar-2026**
**COI**	**None**

The manuscript presents a cross-sectional study aimed at examining key shift attributes and estimating the trade-offs between shift pattern characteristics and pay using a discrete choice experiment (DCE). This is an important and timely research topic. However, several aspects remain unclear and require further clarification and refinement. In particular, the authors are encouraged to follow the STROBE Statement reporting guidelines when presenting the study.

Abstract

1. How were the factors, including shift length, schedule predictability, shift request flexibility, weekend working frequency, pay level, night-shift frequency, and willingness-to-accept (WTA), assessed? It would be helpful to briefly describe the analytical approach in the Methods section of the abstract, as readers may otherwise find it difficult to understand the results presented.
2. The Results section in the abstract should be more concise. It would be preferable to first prioritize the major findings related to the primary outcomes.

Introduction

1. Pay level and satisfaction with pay among shift-working nurses are important issues. Please provide relevant references and discuss this aspect in the introduction. This would help highlight the research gap and strengthen the rationale for the study.
2. Several parts currently presented in the Methods section would be more appropriate in the Introduction. For example, the content on page 4, lines 4–18, and page 5, lines 5–14

appears to describe background information rather than methodological procedures.

3. At the end of the Introduction, the authors should clearly describe the study aims, including the main research objective and the rationale for conducting subgroup analyses, after outlining the research gaps.

Methods

1. In Table 1, how is the night shift defined in this study? Do nurses in the authors' country only work two types of shifts (day and night)? Please clarify and provide more details. In many countries, particularly in Asia, nursing shifts usually include three major shifts (day, evening, and night). Therefore, additional clarification would help international readers better understand the shift patterns used in this study.
2. In addition, Table 1 includes a 9–10-hour shift length (Level 2), where night work is described as a mix of day and night shifts. More explanation is needed, as this configuration may be unfamiliar to readers from other countries.
3. The section describing sample and eligibility is recommended to be separated into two subsections:

   Participants and Setting

   Data Collection / Measures

   The Participants and Setting section should describe the sampling and recruitment process. The Data Collection / Measures section should clearly define all relevant attributes or concepts, including their operational definitions.
4. The statistical analysis software used should be specified.
5. The manuscript contains a large number of abbreviations, which may confuse readers. Please reduce the number of abbreviations and ensure that each abbreviation is fully defined at its first occurrence.

Results

1. The first sentence of the Results section appears more suitable for the Methods or Discussion section.
2. It would be better to present the participant recruitment process, ideally including a flowchart, showing the number of initially approached participants and the final number of included participants (i.e., response rate and completion rate).
3. The categories of independent variables in Table 2 are unclear. For example, the definitions of pay band and bank hours per month should be clarified. These variables should be explicitly described in the Data Collection / Measures section.
4. Table 3 presents the correlation coefficients between the MNL model and shift attributes. However, several variables appear to be categorical variables, such as predictability, shift requests, weekend working, and pay level. It is unclear how correlation coefficients were calculated between the MNL model and these categorical variables. Please clarify the analytical approach, as this could represent a significant methodological concern.

**Reviewer**	**2**
**Name**	**Ameri, Hosein**

Affiliation **Shahid Sadoughi University of Medical Sciences and Health Services**

Date **23-Jun-2026**

COI **None**

The authors investigated how registered nurses' preferences for shift attributes using a DCE. This paper benefits from a well-structured experimental design, and the findings provide valuable insights that could inform future policy decisions regarding workforce retention and staffing management. However, I have some comments as follows:

Introduction

1.The introduction can benefit from a brief sentence justifying why a DCE is the good method for this specific objective. For example, the authors can mention that DCEs allow for the estimation of marginal rates of substitution would strengthen the transition to the methodology.

2.It would be valuable to add one sentence on how current UK policies might be insufficient in addressing the non-monetary preferences of nurses, which this study intends to solve.

Method

3.The study will benefit from clarifying whether the 28 participants in the qualitative phase were sufficient to achieve data saturation regarding the identified attributes.

4.The authors mention the capacity of being traded as a criterion for attribute selection. It is unclear how this capacity was verified. Did the authors conduct a pilot test to ensure that participants perceived these six attributes?

5.Some attributes, particularly Shift Predictability and Shift Requests, may have an overlapping conceptual space. The authors could provide evidence from their pilot testing that respondents were able to clearly distinguish between these two attributes.

6.The authors do not explicitly mention whether a dominance test was included within the 12 choice sets. Given the length of the DCE, it is essential to demonstrate how the authors identified and handled inconsistent respondents. If such a test was absent, the authors should justify how they ensured the internal validity and reliability of the preference data.

7.Given the current standard in DCE applications, could the authors justify why more flexible models, such as Mixed Logit or Latent Class Models, were not employed?

8. Pseudo-R^2 is generally considered a weak metric for model selection compared to information criteria like BIC.

9. Potential position bias in choice tasks. The authors used a MNL model but did not explicitly state whether an alternative specific constant (ASC) was included in the model specification. Given that the attributes are defined as generic shift patterns, the inclusion of

an ASC is generally not required unless there is a hypothesized bias towards one of the unlabeled alternatives. However, if the alternatives were labeled, an ASC is essential to capture unobserved utility associated with the current status.

Results and discussion

10. Since the pay attribute was entered into the model as a categorical variable, using the average of the coefficients to determine the numeraire imposes an assumption of linearity regarding the utility of money. Have you conducted a sensitivity analysis (e.g., considering the pay attribute as a continuous variable) to demonstrate that this averaging choice does not affect the precision of the WTA values.

11. Table 3. The authors performed subgroup analyses to account for workforce heterogeneity. However, it is unclear whether the differences in preferences between these subgroups (e.g., age, pay band, or sector) are statistically significant. I recommend that the authors provide formal statistical tests for the equality of coefficients across subgroups to confirm whether the observed variations are robust and distinct.

12. While the interaction between Mix days/nights * Age is statistically significant, the coefficient itself is quite small. Could the authors discuss the clinical or policy relevance of this interaction? A small change per unit of age suggests that the moderating effect of age may be minimal in practical terms, despite its statistical significance.

13. Several other interactions were not statistically significant. Given the high sample size, the lack of significance here is a strong finding. It suggests that these workforce characteristics do not fundamentally alter how these attributes are valued. I recommend that the authors explicitly state this homogeneity of preference as a key takeaway, although it is indicated.

14. The authors note a significant increase in the aversion to no whole weekends off as the choice tasks progressed. the authors interpret this as preference sharpening, but it could also be interpreted as a fatigue effect or a change in respondent strategy. I suggest the authors further discuss why this specific attribute was sensitive to task order while others were not.

VERSION 1 - AUTHOR RESPONSE

**Reviewer 1**	**Our Response**	**Action Taken**
Abstract: How were the factors, including shift length, schedule predictability, shift request flexibility, weekend working frequency, pay level, night-shift frequency, and willingness-to-accept (WTA), assessed? It would be helpful to briefly describe the analytical approach in the Methods section of the abstract, as readers may otherwise find	We have revised the Methods section of the abstract to briefly describe the analytical approach. We now state that a multinomial logit (MNL) model was used to estimate nurses' relative preferences, and that willingness-to-accept values were derived from the ratio of	Abstract, design section we added “Preferences were estimated using multinomial logit (MNL) models with clustered standard errors; willingness-to-accept (WTA) values were derived as ratios of attribute coefficients to the pay coefficient.”

it difficult to understand the results presented.	attribute coefficients to the pay coefficient.	
Abstract: The Results section in the abstract should be more concise. It would be preferable to first prioritize the major findings related to the primary outcomes.	We have revised the Results section of the abstract to lead with the primary findings (the relative importance of attributes and the key WTA values) before presenting secondary findings. The revised abstract is more concise and prioritises findings related to the primary study objective	Abstract, results section.
Introduction: Pay level and satisfaction with pay among shift-working nurses are important issues. Please provide relevant references and discuss this aspect in the introduction.	We have added a short paragraph to the Introduction discussing pay level and satisfaction with pay among shift-working nurses, supported by relevant references.	Page 3, lines 32-37 we added "Understanding these trade-offs is important because current evidence suggests that pay dissatisfaction is associated with intentions to leave the NHS,[19] and that younger nurses in particular report lower pay satisfaction than their more senior colleagues.[20] Yet it remains unclear whether financial incentives are sufficient to compensate for undesirable rota characteristics, or which aspects of shift work nurses value most highly relative to pay."
Introduction: Several parts currently presented in the Methods section would be more appropriate in the Introduction.	We reviewed the sections identified by the reviewer and strengthened the Introduction to foreground the policy context, the evidence gap, and the rationale for using a DCE. We retained technical details relating to attribute specification and experimental design in the Methods section, where they support transparency and reproducibility.	We have modified both introduction and methods
Introduction: At the end of the Introduction, the authors should clearly describe the study aims, including the main research objective and the rationale for conducting subgroup analyses.	We have revised the final paragraph of the Introduction to state the study aims explicitly. We now specify that the primary objective was to quantify nurses' preferences for shift pattern attributes and	At page 4, lines 42-47 we wrote "The aim of this study is therefore to elicit and quantify registered nurses' stated preferences for key shift pattern attributes and their associated trade-offs, and to

	derive WTA values, and we provide a brief rationale for the prespecified subgroup analyses, noting the a priori hypothesis that preferences would vary by age, pay band, and clinical sector.	derive willingness-to-accept estimates that can inform rota design and enhancement policies. A secondary aim was to examine whether preferences vary across key workforce subgroups including age, seniority, and current shift status, on the basis of the a priori hypothesis that these characteristics are likely to shape the disutility of unsocial working conditions."
Methods: In Table 1, how is the night shift defined in this study? Do nurses only work two types of shifts? Please clarify.	We have added a footnote to Table 1 clarifying that, in line with standard NHS practice, a night shift is any shift that includes at least three hours of work between 11pm and 6am.	Table 1, "Note: Night shifts are defined any shift that includes at least three hours of work between 11pm and 6am, consistent with standard NHS Agenda for Change terms."
Methods: Table 1 includes a 9–10-hour shift length; more explanation is needed as this configuration may be unfamiliar.	This explanation is provided below, page 5, Lines "We included shift length with three levels to evaluate trade-offs between short, mid-length and very long duties. The evidence questions the value propositions of ≥12-hour shifts (fatigue, productivity/safety concerns),9 so estimating preferences for alternatives is policy-relevant. A 9–10 h option was added because DCEs are designed to test plausible alternatives that may not yet be widespread, and recent research found that, while seldom worked, 9-10 hours was the ideal shift length for 45% of the 832 registered nurses in the sample.13"	N/A
Methods: The section describing sample and eligibility should be separated into Participants and Setting and Data Collection / Measures.	We have restructured the Methods section as requested, separating the description of participants and eligibility criteria from the data collection subsection	See page 6, line 129 and page 7, line 136
Methods: The statistical analysis software used should be specified.	We have added a statement to the statistical analysis subsection specifying that all analyses were conducted in R	At page 8, Lines 192-193 we added "All analyses were conducted in R, version 4.5.0[34] and the apollo package.[35]"

Methods: The manuscript contains a large number of abbreviations; please reduce and define them.	We have retained only the following abbreviations: DCE, WTA, MNL	Throughout methods, we have removed unnecessary abbreviations
Results: The first sentence appears more suitable for Methods or Discussion.	We have relocated the opening sentence of the Results section to the Methods section where it more appropriately belongs.	At page 7, Line 147-148 we moved "The median response time was 7 minutes and 12 seconds."
Results: Present the participant recruitment process including response rate.	The recruitment process is detailed in methods, and we added the response rate in the results section	At page 8, Line 206-207 we added "The final sample comprised 1,449 registered nurses, yielding a response rate of 2.9%. No respondents were excluded after completing the DCE tasks."
Results: The categories of independent variables in Table 2 are unclear.	We have clarified both in methods and in the table that pay band refers to NHS Agenda for Change salary band. Bank hours per month refers to additional paid shifts undertaken through staff bank arrangements, beyond contracted hours.	Table 2 note "Pay band refers to NHS Agenda for Change salary band. Bank hours per month refers to additional paid shifts undertaken through staff bank arrangements, beyond contracted hours."
Results: Table 3 presents correlation coefficients; please clarify method for categorical variables.	We note that Table 3 presents multinomial logit coefficients, not correlation coefficients. We have clarified this in the table title and accompanying text to avoid confusion. Coefficients for categorical variables represent the utility of each level relative to the reference category, as is standard in MNL models.	At page 11, Lines 229-231 we wrote "The coefficients reported are MNL utility weights; each reflects the estimated change in utility associated with a given attribute level relative to the reference category (the most preferred level for each attribute)."
**Reviewer 2**		
Introduction: .The introduction can benefit from a brief sentence justifying why a DCE is the good method for this specific objective. For example, the authors can mention that DCEs allow for the estimation of marginal rates of substitution would strengthen the transition to the methodology.	We have added a sentence to the Introduction noting that DCEs are particularly suited to this objective because they allow the simultaneous estimation of preferences across multiple attributes, quantification of the relative importance of those attributes, and derivation of marginal rates of substitution, including willingness-to-accept monetary compensation for non-monetary working conditions	At page 3, lines 37-41 we added "Discrete choice experiments (DCEs) are well suited to quantifying these monetary and non-monetary trade-offs simultaneously, because they allow estimation of the relative importance of multiple attributes and derivation of marginal rates of substitution including willingness-to-accept monetary compensation for non-monetary working conditions."

Introduction: It would be valuable to add one sentence on how current UK policies might be insufficient in addressing the non-monetary preferences of nurses, which this study intends to solve.	We have added a sentence to the Introduction noting that while recent NHS workforce policy has focused predominantly on non-monetary preferences of nurses, there is limited evidence on how non-monetary aspects of working conditions, such as scheduling flexibility and shift predictability, are valued by nurses, and that this evidence gap motivates the present study.	At page 3, lines 19-23 we added "Although recent NHS workforce policies have increasingly recognised the importance of flexible working and staff experience,18 19 relatively little quantitative evidence exists on how nurses value specific rota characteristics, such as predictability, flexibility and weekend working, or the trade-offs they are willing to make between these factors and pay."
Methods: The study will benefit from clarifying whether the 28 participants in the qualitative phase were sufficient to achieve data saturation regarding the identified attributes.	We have added a statement to the Methods section clarifying that data saturation was assessed iteratively during the qualitative phase. No new attributes or themes emerged in the final interviews, and the research team judged that saturation had been reached. This is consistent with recommendations for qualitative attribute development in discrete choice experiments, which advocate iterative data collection and analysis until no new relevant attributes or concepts emerge	At page 4, Lines 66-70 we added "Data saturation was assessed iteratively throughout data collection and analysis. No new shift-pattern attributes or concepts relevant to rota preferences were identified during the final interviews. This approach is consistent with recommendations for qualitative attribute development in discrete choice experiments."
Method (Reviewer 2): The authors mention the capacity of being traded as a criterion for attribute selection. It is unclear how this capacity was verified. Did the authors conduct a pilot test to ensure that participants perceived these six attributes?	We thank the reviewer for this comment. By "capacity to be traded", we refer to whether attributes could realistically vary across shift patterns and be considered by respondents in relation to other rota characteristics. Candidate attributes were identified through qualitative interviews and literature review and were retained only if they were judged to be salient, modifiable through rota design, and capable of varying independently from other attributes. Tradeability was further explored during pilot testing, where participants	At page 4, Lines 70-79 we wrote "Our aim was to include attributes that managers can feasibly adjust, that are relevant to nurses, that capture the main trade-offs inherent in shift work and that have the capacity of being traded. During attribute development, we considered whether candidate attributes were salient to participants, amenable to organisational change, and capable of being traded against alternative rota characteristics within realistic work scenarios. Tradeability was assessed during qualitative interviews and pilot

	were asked to complete the choice tasks and provide feedback on attribute interpretation and decision-making. No concerns were identified regarding respondents' ability to consider trade-offs between the selected attributes. We have clarified this process in the Methods section.	testing by exploring participants' interpretations of the attributes and whether they considered them when making choices between hypothetical shift patterns. No substantial concerns regarding attribute relevance, overlap, or inability to make trade-offs were identified."
Method: Some attributes, particularly Shift Predictability and Shift Requests, may have an overlapping conceptual space. The authors could provide evidence from their pilot testing that respondents were able to clearly distinguish between these two attributes	We acknowledge that Shift Predictability and Shift Requests are conceptually adjacent, as both relate to the degree of control nurses have over their schedules. However, they capture distinct aspects: Shift Predictability refers to how far in advance shift schedules are communicated, while Shift Requests refers to the capacity to influence which shifts are assigned. In our qualitative phase, participants consistently distinguished between these two dimensions when discussing their working conditions. We have added a sentence to the Methods section making this distinction explicit	At page 5, Lines 79-85 we wrote "The potential for conceptual overlap between Shift Predictability and Shift Requests was carefully considered. Shift Predictability refers to how far in advance shift schedules are communicated, while Shift Requests refers to the capacity to influence which shifts are assigned. In the qualitative phase, participants consistently distinguished between these two dimensions when discussing their working conditions: nurses described predictability as knowing what was coming, and requesting as having a say in shaping it. Both were retained as conceptually and practically distinct attributes."
Method: The authors do not explicitly mention whether a dominance test was included within the 12 choice sets. Given the length of the DCE, it is essential to demonstrate how the authors identified and handled inconsistent respondents. If such a test was absent, the authors should justify how they ensured the internal validity and reliability of the preference data.	We thank the reviewer for this comment. A formal dominance task was not included. Consequently, we could not screen individual respondents for dominated choices or individual-level non-trading behaviour. However, coefficient signs and magnitudes were consistent with theoretical expectations, and task-order analyses suggested broadly stable preferences for key attributes. We have acknowledged the absence of a dominance test as a limitation.	At page 19, Lines 413-415 we added "A formal dominance or rationality task was not included, meaning that individual-level dominated choices or non-trading behaviour could not be directly assessed."

Method: Given the current standard in DCE applications, could the authors justify why more flexible models, such as Mixed Logit or Latent Class Models, were not employed?	Thanks for this important methodological question. We chose MNL as our primary model for two reasons. First, MNL provides a transparent and interpretable baseline that facilitates communication of findings to a policy and practitioner audience. Second, we addressed preference heterogeneity through prespecified subgroup analyses and interaction terms, which we judged to be more aligned with our policy-focused objectives than the statistical decomposition of heterogeneity provided by Mixed Logit random parameters or Latent Class membership. We have added a paragraph to the Methods section making this rationale explicit, and acknowledge that Mixed Logit or Latent Class approaches could be used in future work to further characterise heterogeneity	At page 7, lines 156-162 we added "MNL was selected as the primary model because the objective was to estimate transparent, policy-interpretable average preferences and WTA benchmarks. Observed heterogeneity was explored through prespecified subgroup and interaction analyses. We acknowledge that MNL assumes independence of irrelevant alternatives and homogeneous unobserved preferences; mixed logit and latent class models would relax these assumptions and represent a valuable extension, but were beyond the policy-focused scope of the present analysis."
Method: Pseudo-R^2 is generally considered a weak metric for model selection compared to information criteria like BIC.	We agree that pseudo-R^2 has limitations as a model fit metric. We have added BIC and AIC values to the model outputs	At page 8, lines 191-193 we wrote: "Model diagnostics included examination of coefficient signs and magnitudes, confidence interval widths, and information criteria (BIC, AIC). "We added AIC and BIC at the bottom of tables, and at page 17, lines 327-334 we wrote "Nested task-position models were compared using likelihood-ratio tests and information criteria. Adding a linear task-position term improved model fit compared with the baseline model (LR = 23.67, df = 1, $p < 0.001$; AIC 20431.81 vs 20453.48; BIC 20505.71 vs 20522.10). Adding attribute-by-task interactions also improved fit relative to baseline (LR = 24.38, df = 3, $p < 0.001$), although the

		improvement was modest once additional parameters were penalised (AIC 20435.10; BIC 20519.55). These findings suggest a small task-position effect, but do not indicate substantial instability in preferences across the DCE.”
Method: Potential position bias in choice tasks. The authors used a MNL model but did not explicitly state whether an alternative specific constant (ASC) was included in the model specification. Given that the attributes are defined as generic shift patterns, the inclusion of an ASC is generally not required unless there is a hypothesized bias towards one of the unlabeled alternatives. However, if the alternatives were labeled, an ASC is essential to capture unobserved utility associated with the current status.	We thank the reviewer for this comment. The alternatives were unlabelled generic shift patterns (Job A and Job B). An alternative-specific constant for Job A was included to assess potential position bias. The ASC was small and not statistically significant ($\beta = 0.03$, $p = 0.06$), suggesting limited evidence of systematic left-right choice bias. We have clarified this in the Statistical Analysis section.	At page 7, lines 163-166 we wrote<br>“The choice alternatives were unlabelled generic shift patterns, presented as Job A and Job B. An alternative-specific constant (ASC) for Job A was included as a position constant to assess any systematic preference for one presented alternative over the other that was not explained by the observed attributes.”<br>And at page 11, Lines 231-233 we wrote “The ASC for Job A was small and not statistically significant ($\beta = 0.03$, $p = 0.06$), indicating limited evidence of systematic position bias.”
Results/Discussion: Since the pay attribute was entered into the model as a categorical variable, using the average of the coefficients to determine the numeraire imposes an assumption of linearity regarding the utility of money. Have you conducted a sensitivity analysis (e.g., considering the pay attribute as a continuous variable) to demonstrate that this averaging choice does not affect the precision of the WTA values.	Thank you for this suggestion. We undertook two complementary sensitivity analyses to assess the robustness of the WTA estimates.<br><br>First, we recalculated WTA values using alternative monetary numeraires based on the separate pay-level coefficients within the primary categorical-pay model, rather than the average pay coefficient used in the main analysis. Although the absolute magnitude of some WTA estimates changed, the relative ordering of attributes remained broadly unchanged and the same job characteristics consistently emerged as the most important drivers of preferences.	At page 13, lines 263-273 we added “Sensitivity analyses demonstrated that the WTA findings were robust to alternative specifications of the pay attribute. Recalculating WTA values using alternative pay-level coefficients resulted in some variation in absolute compensation estimates but did not materially alter the ordering of preferences. Similarly, a model specifying pay as a continuous attribute produced near-identical model fit to the primary specification (AIC 20453.46 vs 20453.48; BIC 20516.81 vs 20522.10). Across both sensitivity analyses, nights-only working and having no whole weekends off remained the two most strongly disliked

	Second, we estimated an additional sensitivity model in which pay was specified as a continuous attribute coded in 10 percentage-point increments. Model fit was virtually identical to the primary model (AIC 20453.46 vs 20453.48; BIC 20516.81 vs 20522.10), and the substantive interpretation of the results was unchanged. In both specifications, nights-only working and having no whole weekends off remained the attributes associated with the greatest disutility. The ranking of attribute importance was highly consistent across models, indicating that the conclusions are robust to alternative specifications of the pay attribute.	job characteristics, followed by mixed day/night working. These findings suggest that the substantive interpretation of WTA estimates is robust to alternative pay specifications. Results from an additional sensitivity analysis specifying pay as a continuous attribute are presented in Supplementary Tables S1a and S1b"
Results/Discussion: Table 3. The authors performed subgroup analyses to account for workforce heterogeneity. However, it is unclear whether the differences in preferences between these subgroups (e.g., age, pay band, or sector) are statistically significant. I recommend that the authors provide formal statistical tests for the equality of coefficients across subgroups to confirm whether the observed variations are robust and distinct.	In response to the reviewer's suggestion, we formally tested subgroup differences using z-tests of coefficient equality. These analyses confirmed significant heterogeneity by age and current shift status, with younger nurses demonstrating significantly greater sensitivity to pay reductions and loss of whole weekends off than older nurses, and nurses not currently working shifts showing significantly greater aversion to nights-only working and intensive weekend working than current shift workers. Whilst some coefficient differences were observed across pay bands, only aversion to nights-only working differed significantly between Band 5 and Band 8+ nurses; differences in pay sensitivity were not statistically significant.	At page 14, lines 284-295 we added "Whilst Table 5 presents subgroup-specific coefficients, formal tests of coefficient equality were conducted to determine whether observed differences between groups were statistically significant (Supplementary Table 1). Significant differences were observed by age and current shift status. Younger nurses were significantly more sensitive to pay reductions than older nurses ($z = -5.29$, $p < 0.001$) and were more averse to having no whole weekends off ($z = -4.54$, $p < 0.001$). Nurses not currently working shifts were significantly more averse than current shift workers to nights-only working ($z = 7.90$, $p < 0.001$) and to having no whole weekends off ($z = 4.25$, $p < 0.001$). Senior nurses (Band 8+) were also

		significantly more averse to nights-only working than Band 5 nurses (z = 2.74, p = 0.006). In contrast, although coefficients differed numerically across pay bands, formal tests did not support statistically significant differences in pay sensitivity between Band 5 and Band 8+ respondents (z = 1.53, p = 0.126).” And we added Supplemenatary table 2. And in Discussion we added at page 17, lines 346-347 “subgroup analysis revealed the strongest evidence of heterogeneity by age and current shift status, with more limited evidence of pay-band differences”
Results/Discussion: While the interaction between Mix days/nights * Age is statistically significant, the coefficient itself is quite small. Could the authors discuss the clinical or policy relevance of this interaction? A small change per unit of age suggests that the moderating effect of age may be minimal in practical terms, despite its statistical significance.	We thank the reviewer for this observation. We agree that the per-year interaction coefficient is small and have revised the Discussion to distinguish statistical from practical significance. We note that the coefficient represents a marginal effect per year of age; therefore, although the annual change is modest, the cumulative effect across the age range of the nursing workforce is larger. We have revised the manuscript to emphasise that this finding should be interpreted as evidence of a gradual shift in preferences with age rather than a large effect at the individual-year level.	At page 18, lines 388-390 we added "Although the interaction between age and mixed day/night working was statistically significant, its magnitude was small, indicating that age-related differences in preferences emerge gradually rather than representing a large practical effect at the individual level.”
Results/Discussion: Several other interactions were not statistically significant. Given the high sample size, the lack of significance here is a strong finding. It suggests that these workforce characteristics do not fundamentally alter how these attributes are valued. I recommend that the authors explicitly	We agree with the reviewer's framing. With a sample of 1,449 and adequate statistical power, the absence of significant interactions for the majority of tested subgroup characteristics is a substantively meaningful finding, not merely a null result. It suggests that preferences for	At page 17, lines 347-353 we added “Third, the dominant preference pattern was highly consistent across the nursing workforce: intensive night work and loss of whole weekends were disliked across demographic and employment groups. This suggests that

state this homogeneity of preference as a key takeaway, although it is indicated.	shift pattern attributes are broadly consistent across the nursing workforce. We have elevated this as an explicit conclusion in the Discussion	these rota features are core workforce concerns rather than issues confined to specific subgroups. Interaction analyses showed limited evidence of effect modification, with only two moderating effects: a gradual age-related increase in aversion to mixed day/night rotations, and greater aversion to 12.5-hour shifts among staff with longer commutes."
Results/Discussion: The authors note a significant increase in the aversion to no whole weekends off as the choice tasks progressed. the authors interpret this as preference sharpening, but it could also be interpreted as a fatigue effect or a change in respondent strategy. I suggest the authors further discuss why this specific attribute was sensitive to task order while others were not.	We have revised the Results and Discussion to avoid over-interpreting this effect. We now state that the strengthening of aversion to losing whole weekends off across tasks may reflect preference sharpening, fatigue, or strategic updating, and that these mechanisms cannot be definitively disentangled with the current design.	At page 19, lines 411-415 we added "Finally, the intensification of aversion to weekend working across tasks may reflect preference sharpening, fatigue, or strategic updating, and these mechanisms cannot be definitively disentangled with the current design."

VERSION 2 - REVIEW

Reviewer 2

Name Ameri, Hosein

Affiliation Shahid Sadoughi University of Medical Sciences and Health Services

Date 23-Jul-2026

COI

Thank you for your responses. I believe the current version can move forward.
