# Supplementary material for: Balancing the rota: registered nurses’ preferences for shift patterns – a discrete choice experiment
Source: BMJ Open. 2026 Aug 18;16(8):e116968. doi: 10.1136/bmjopen-2026-116968 (PMC13504907; doi:10.1136/bmjopen-2026-116968)
Supplement: Supplementary file 2 [file bmjopen-16-8-s001.docx]

**Supplementary Table S1a.** Sensitivity analysis of WTA estimates using alternative monetary numeraires

| **Attribute** | **Main analysis (average pay coefficient)** | **Sensitivity 1 (Pay coefficient level 1)** | **Sensitivity 2 (Pay coefficient level 2)** |
| --- | --- | --- | --- |
| Very high night shifts (vs low) | 2.94 | 2.12 | 4.79 |
| High weekend frequency (vs low) | 2.08 | 1.50 | 3.40 |
| High night shifts (vs low) | 1.17 | 0.84 | 1.90 |
| Medium weekend frequency (vs low) | 0.74 | 0.53 | 1.20 |
| Unpredictable schedule (vs predictable) | 0.58 | 0.42 | 0.95 |
| Limited shift choices level 1 (vs most flexible) | 0.50 | 0.36 | 0.81 |
| Weekly hours level 1 (vs level 3) | 0.47 | 0.34 | 0.77 |
| Limited shift choices level 2 (vs most flexible) | 0.26 | 0.19 | 0.42 |
| Sometimes predictable (vs predictable) | 0.07 | 0.05 | 0.12 |
| Weekly hours level 2 (vs level 3) | -0.38 | -0.28 | -0.62 |

Note: Values are presented as absolute WTA magnitudes for ease of interpretation. Sensitivity analyses recalculated willingness-to-accept (WTA) using (i) the average of the two pay coefficients used in the primary analysis, (ii) the coefficient associated with pay level 1 only, and (iii) the coefficient associated with pay level 2 only. Although the magnitude of WTA estimates varied across specifications, the relative ordering of attributes remained unchanged.

**Supplementary Table S1b. Sensitivity analysis of willingness-to-accept estimates using a continuous pay specification**

| **Attribute level** | **WTA (10% pay increments)** | **Equivalent percentage pay increase (%)** |
| --- | --- | --- |
| Nights only | 4.26 | 42.65 |
| No whole weekends off | 3.01 | 30.12 |
| Mixed day and night shifts | 1.69 | 16.93 |
| One whole weekend off per month | 1.07 | 10.72 |
| Unpredictable schedule | 0.85 | 8.54 |
| Only shift swaps accepted | 0.74 | 7.36 |
| 12.5-hour shifts | 0.70 | 7.04 |
| Two scheduling requests per month | 0.40 | 4.00 |
| Sometimes predictable schedule | 0.13 | 1.27 |
| 9–10 hour shifts | -0.55 | -5.48 |

Notes: Sensitivity analysis in which the pay attribute was specified as a continuous variable coded in 10 percentage-point increments rather than as categorical pay levels. WTA estimates represent the percentage pay increase required to compensate respondents for a less desirable job characteristic. Negative values indicate a preferred attribute level relative to the reference category. Model fit was virtually identical to the primary specification (AIC 20453.46 vs 20453.48; BIC 20516.81 vs 20522.10), and the ordering of attribute importance was unchanged, with nights-only working and having no whole weekends off remaining the most strongly disliked job characteristics
