# Supplementary material for: Balancing the rota: registered nurses’ preferences for shift patterns – a discrete choice experiment
Source: BMJ Open. 2026 Aug 18;16(8):e116968. doi: 10.1136/bmjopen-2026-116968 (PMC13504907; doi:10.1136/bmjopen-2026-116968)
Supplement: Supplementary file 3 [file bmjopen-16-8-s002.docx]

Supplementary Table S2 Formal tests of coefficient equality across selected respondent subgroups

| Comparison | Attribute | Difference in coefficient | z-statistic | p-value |
| --- | --- | --- | --- | --- |
| Younger (<40 years) vs Older (≥55 years) | No whole weekends off | -0.341 | -4.54 | <0.001 |
| Younger (<40 years) vs Older (≥55 years) | No pay increase | -0.378 | -5.29 | <0.001 |
| Current shift workers vs Non-shift workers | Nights only | 0.818 | 7.90 | <0.001 |
| Current shift workers vs Non-shift workers | No whole weekends off | 0.329 | 4.25 | <0.001 |
| Band 5 vs Band 8+ | Nights only | 0.773 | 2.74 | 0.006 |
| Band 5 vs Band 8+ | No pay increase | 0.249 | 1.53 | 0.126 |

*Notes:* z-tests were conducted using the difference in coefficients divided by the square root of the summed squared standard errors from the separate subgroup models. Positive z-statistics indicate that the coefficient was larger in magnitude for the second group listed, whereas negative z-statistics indicate that the coefficient was larger in magnitude for the first group listed.
